# Vitamin D supplementation positively affects anthropometric indices: Evidence obtained from an umbrella meta-analysis

**DOI:** 10.3389/fnut.2022.980749

**Published:** 2022-09-07

**Authors:** Vali Musazadeh, Meysam Zarezadeh, Faezeh Ghalichi, Fateme Hamedi Kalajahi, Zohreh Ghoreishi

**Affiliations:** ^1^Student Research Committee, Tabriz University of Medical Sciences, Tabriz, Iran; ^2^Department of Community Nutrition, School of Nutrition and Food Science, Tabriz University of Medical Sciences, Tabriz, Iran; ^3^Nutrition Research Center, School of Nutrition and Food Sciences, Tabriz University of Medical Sciences, Tabriz, Iran

**Keywords:** vitamin D, anthropometric indices, obesity, body mass index, umbrella meta-analysis

## Abstract

Despite the growing evidence from meta-analyses on vitamin D’s anti-obesity properties, their results are controversial. The current umbrella review was performed to assess the available evidence and provide a conclusive explanation in this regard. The international databases PubMed, Scopus, Embase, Web of Science and Google Scholar were systematically searched till March, 2022. A random-effects model was used to run the meta-analysis. All meta-analyses that examined the effect of vitamin D supplementation on BW, BMI, WC, and fat mass were included. Findings of 14 meta-analyses revealed that vitamin D supplementation reduced body mass index (BMI) (ES: −0.11 kg/m^2^; 95% CI: −0.18, −0.05, *p*?0.001; *I*^2^ = 61.0%, *p* < 0.001), and waist circumference (WC) (ES = −0.79 cm; 95% CI: −1.20, −0.37; *p* < 0.001; *I*^2^ = 46.5%, *p* = 0.096) in comparison to control group. However, the effects of vitamin D on body weight (ES = −0.16 kg, 95% CI: −0.36, 0.04; *p* = 0.125; *I*^2^ = 57.0%, *p* = 0.017), and fat mass (ES: 0.02, 95% CI: −0.20, 0.24, *p* = 0.868; *I*^2^ = 0.0%, *p* = 0.531) were not considerable. Vitamin D supplementation significantly improved levels of obesity indices such as BMI, and WC.

## Introduction

According to the World Health Organization definition, a body mass index (BMI) of ≥ 30 kg/m^2^ is considered as obesity which currently is a global public health concern. Cardiovascular disease, stroke, type 2 diabetes mellitus (T2DM) and certain types of cancer as the leading causes of preventable death, are obesity-related conditions. According to data from 2018 to 2019 from the National Health and Nutrition Examination Survey (NHANES), almost 1 in 3 adults (30.7%) are overweight, more than 2 in 5 adults (42.4%) are obese, and about 1 Out of every 11 adults (9.2%) have severe obesity. By another mean, more than 2.5 billion adult people are overweight and obese worldwide (1, 2).

The Obesity studies emphasize the link between the metabolism of macronutrients and deficiency of serum vitamins levels with weight gain. Fat-soluble vitamins are required for various actions and prevention of diseases (3). Vitamin D as a fat soluble vitamin not only is involved in calcium and phosphorus homeostasis, and bone metabolism, but also plays an important role in regulating collagen type 1 production, muscle function, cell differentiation, insulin secretion, and the immune system (4). Some chronic disorders such as insulin resistance, metabolic syndrome, atherosclerosis, neurodegenerative diseases and obesity are associated with vitamin D deficiency (5–7). An inverse association between adipose tissue and obesity degree with serum levels of vitamin D has been observed in studies (8–10). Cheng et al. reported that vitamin D deficiency was three times higher in subjects with high body fat concentration (7). More fat concentration in the body causes the storage of more vitamin D in adipose tissue. Vitamin D volume dilution mechanism that has been considered by researchers today, suggested that vitamin D is distributed in muscles, fat, and liver. With obesity the amount of vitamin D are increased in these tissues, resulting in lower serum levels of vitamin D (11, 12). Another possible mechanism is increased lipogenesis in vitamin D deficiency condition. Elevated parathyroid hormone secretion in vitamin D deficiency results in more influx of calcium into the adipocyte, stimulating lipogenesis (13). Several clinical trial studies investigated effect of vitamin D supplementation on anthropometric indices in obese people. While some studies have shown a positive effect of supplementation on body fat and body mass index, some other studies have reported no effect of vitamin D supplementation on total body fat (14–16).

The results of the meta-analysis are also contradictory in this regard. While some meta-analysis studies show a positive effect of the vitamin D supplement on weight loss and waist circumference (17, 18), some have reported ineffectiveness of the supplementation on anthropometric indices (19, 20). Therefore, we conducted present umbrella meta-analysis to clarify the effect of vitamin D supplementation on anthropometric indices in different health conditions.

## Methods

### Literature search

Google Scholar, PubMed, Scopus, and Web of Science were systematically searched from the earliest date available up to March, 2022. The study implementation of the study was finished on June 2022. The used MeSH terms and keywords include: (“vitamin d”OR “ergocalciferols”) AND (treatment) OR (supplementation) OR (vitamin d3) OR (vitamin d2) OR (intake) AND (“Body composition”) OR (“Weight Loss”) OR (“body weight”) OR (“body weight changes”) OR (“body mass index”) OR (obesity) OR (“body weight”) OR (BMI)[OR (“waist circumference”) OR (WC) OR (“fat mass”) OR (“lean mass”) AND (“systematic review”) OR (“meta-analysis”). In addition, a manual search of the references of eligible studies was done to minimize the risk of missing relevant papers. PRISMA guidelines were followed during implementation of all steps of this study.

### Inclusion and exclusion criteria

PICO criteria for current umbrella meta-analysis were as follows: Population/Patients (P: adults, 18-year-old or above, who were treated with vitamin D); Intervention (I: vitamin D); Comparison (C: control or placebo group); Outcome (O: obesity indices including body weight, BMI, waist circumference (WC) and fat mass. Meta-analysis studies in English examining the effects of vitamin D supplementation on anthropometric indices including body weight, BMI, WC, and fat mass which have reported the effect sizes (ES) and corresponding confidence intervals (CI) were considered eligible. Original studies, editorials, letters to the editor, and studies with low-quality score were excluded.

### Study selection and data extraction

Two reviewers (VM, FHK) conducted the process of screening and inclusion of articles independently. Additionally, the reference lists of all included studies were screened for eligible studies. Following data were extracted from the included meta-analyses: first author’s name, publication year, country, type of vitamin D, supplementation dosage, duration range of supplementation, study population, number of participants, participant demographics, and main results [effect size with 95% confidence intervals (95% CIs)]. Any disagreements were resolved by discussion with the third reviewer (MZ).

### Methodological quality and assessment of the certainty

The Assessment of Multiple Systematic Reviews 2 (AMSTAR2) tool was employed to evaluate the methodological quality of systematic reviews and meta-analyses by two reviewers (VM, FHK), independently. AMSTAR2 questionnaire contains 16 items asking reviewers to respond with a “Yes” or “Partial Yes” or “No” or “No Meta-analysis” option. According to the AMSTAR2 checklist, quality was classified as “Critically low quality,” “low quality,” “moderate quality,” and “high quality” (21). We evaluated the overall certainty of the evidence of the included meta-analyses using GRADE tools. The quality of evidence was classified into four categories based on evaluation criteria, i.e., high, moderate, low, and very low (22).

### Data synthesis and statistical analysis

ESs and CIs were used to calculate the overall effect sizes. We assessed data in terms of heterogeneity for each pooled analysis based on Cochrane Q test and *I*^2^ statistics. *I*^2^-values of 25, 50, and 75% were categorized as low, moderate, and high heterogeneity, respectively (23). *I*^2^-value > 50% or *p* < 0.1 for the *Q*-test were considered as significant heterogeneity among the studies. Random-effects model was used to conduct the meta-analysis. To find the possible sources of heterogeneity, subgroup analyses based on age, sample size, dose, duration, type of effect size, gender, and health condition were performed. To determine the overall effect size dependence on a specific study, sensitivity analysis was utilized. The small-study effect evaluated by the Begg’s and Egger’s tests. Also, publication bias was assessed by visual inspection of the funnel plots whenever the number of included studies were ≥ 10. Trim and fill analysis was conducted where the publication bias was present. Version 16 of Stata (Stata Corporation, College Station, TX, United States) was used to perform the analysis in this study. *P*-value < 0.05 was considered as significant level.

## Results

### Study selection and study characteristics

**FIGURE 1:**
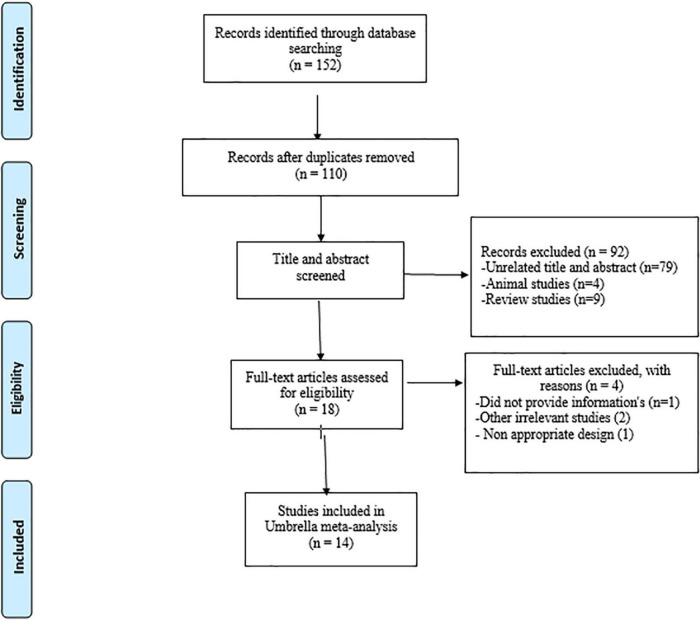
Flowchart of the study selection.

PRISMA flow diagram is shown in Figure 1. We found 152 articles after searching electronic databases. Total number of 42 duplicate articles were removed. Then the remaining 110 articles were screened carefully by title and abstract. Among those, full-text of 18 articles was evaluated. At the end, 14 meta-analyses were included in the current umbrella review. An overview of the characteristics of qualified articles is shown in Table 1. The included studies were published from 2013 to 2021, and the mean age of participants was between 27 and 62 years. The average amount of administered vitamin D among studies was between 1,000 and 59,000 IU/day. The duration of vitamin D supplementation ranged from 8 to 43 weeks. The location of the studies were as follows: four in China (20, 24–26), two in Iran (18, 27), two in United States (28, 29), one each in Philippines (30), Bahrain (17), India (19), Australia (31), United Kingdom (32), and Brazil (33). Table 1 describes the quality of randomized controlled trials that were included in the included meta-analyses.

**TABLE 1:** Study characteristics of included studies.

References	No. of studies in meta-analysis	Location duration	No. of participants in meta-analysis	Age (year)	Dose (IU/day)	Quality assessment scale and outcome
Guevara et al. (30)	11	Philippines 8 week	NR	46.5	NR	NR
Duan et al. (24)	20	China 37 week	3,153	42	2,800	Yes (cochrane) 11/20 high
Miao et al. (25)	11	China 14 week	483	27	5,768	Yes (cochrane) 8/11 high
Rezaei et al. (18)	16	Iran 15 week	1,115	NR	59,000	Yes (cochrane) 7/7 high
Perna (17)	11	Bahrain 15 week	947	38	6,588	Yes (cochrane) 7/11 high
Mora et al. (28)	9	United States 43 week	1,683	48	2,150	Yes (jadad) 8/9 high
Chandler et al. (29)	12	United States 29 week	4,239	62	2,800	Yes (cochrane) 10/12 high
Manousopoulou et al. (32)	5	United Kingdom 24 week	1,328	46	6,200	Yes (jadad) 5/5 high
Saha and Saha (19)	11	India 8 week	857	30	2,300	Yes (cochrane) 10/11 high
Tabriz et al. (27)	7	Iran 11.5 week	332	45	3,000	Yes (cochrane) 7/7 high
Pathak et al. (31)	12	Australia 24 week	1,210	39	16,000	Yes (Jadad) 12/12 high
Kron-Rodrigues et al. (33)	4	Brazil 12 week	174	NR	1,000	Yes (cochrane) 3/4 high
Wang et al. (20)	10	China 11 week	115	28	5,900	Yes (cochrane) 8/10 high
Zou et al. (26)	24	China 16 week	1,932	54	23,000	Yes (cochrane) 15/24 high

### Methodological quality

The results of the AMSTAR2 questionnaire are presented in Table 2. Eleven of the papers were of high quality, two were moderate, and one was critically low quality. All quality outcomes were estimated as moderate using the GRADE system (based on indirectness) (Table 3).

**TABLE 2:** Results of assess the methodological quality of meta-analysis.

Study	Q1*	Q2	Q3	Q4	Q5	Q6	Q7	Q8	Q9	Q10	Q11	Q12	Q13	Q14	Q15	Q16	Quality assessment
Guevara et al. (30)	No	No	Yes	Partial yes	No	No	Yes	No	Yes	No	Yes	No	No	No	No	No	Critically low
Duan et al. (24)	No	Yes	Yes	Yes	Yes	Yes	Yes	Yes	Yes	No	Yes	Yes	Yes	Yes	Yes	Yes	High
Miao et al. (25)	No	Partial yes	Yes	Partial yes	Yes	Yes	Yes	Yes	Yes	No	Yes	Yes	Yes	Yes	Yes	Yes	High
Rezaei et al. (18)	Yes	Yes	Yes	Yes	Yes	Yes	Yes	Yes	Yes	Yes	Yes	Yes	Yes	Yes	Yes	Yes	High
Perna (17)	No	Partial yes	Yes	Yes	Yes	Yes	Yes	Yes	Yes	No	Yes	Yes	Yes	Yes	NO	Yes	High
Mora et al. (28)	No	Yes	Yes	Yes	Yes	Yes	Yes	Partial yes	No	No	Yes	Yes	No	Yes	Yes	No	Moderate
Chandler et al. (29)	Yes	Yes	Yes	Yes	Yes	Yes	Yes	Yes	Yes	No	Yes	Yes	Yes	Yes	Yes	No	High
Manousopoulou et al. (32)	Yes	Yes	Yes	Yes	Yes	Yes	Yes	Yes	Yes	Yes	Yes	Yes	Yes	Yes	Yes	Yes	High
Kron-Rodrigues et al. (33)	Yes	Yes	Yes	Yes	Yes	Yes	Yes	Yes	Yes	Yes	Yes	Yes	Yes	Yes	No	Yes	High
Saha and Saha (19)	No	Partial yes	Yes	Yes	Yes	Yes	Yes	Yes	Yes	No	Yes	Yes	No	Yes	Yes	Yes	High
Tabriz et al. (27)	No	Partial yes	Yes	Yes	Yes	Yes	Yes	Yes	Yes	No	Yes	Yes	No	Yes	Yes	Yes	High
Pathak et al. (31)	No	Partial yes	Partial yes	Yes	Yes	Yes	Yes	Yes	Yes	No	Yes	Yes	No	No	Yes	Yes	Moderate
Wang et al. (20)	No	Partial yes	Yes	Partial yes	Yes	Yes	Yes	Yes	Yes	Yes	Yes	Yes	No	Yes	Yes	Yes	High
Zou et al. (26)	No	Yes	Yes	Yes	Yes	Yes	Yes	Yes	No	Yes	Yes	Yes	Yes	Yes	Yes	Yes	High

*1. Did the research questions and inclusion criteria for the review include the components of PICO? 2. Did the report of the review contain an explicit statement that the review methods were established prior to the conduct of the review and did the report justify any significant deviations from the protocol? 3. Did the review authors explain their selection of the study designs for inclusion in the review? 4. Did the review authors use a comprehensive literature search strategy? 5. Did the review authors perform study selection in duplicate? 6. Did the review authors perform data extraction in duplicate? 7. Did the review authors provide a list of excluded studies and justify the exclusions? 8. Did the review authors describe the included studies in adequate detail? 9. Did the review authors use a satisfactory technique for assessing the risk of bias (RoB) in individual studies that were included in the review? 10. Did the review authors report on the sources of funding for the studies included in the review? 11. If meta-analysis was performed, did the review authors use appropriate methods for statistical combination of results? 12. If meta-analysis was performed, did the review authors assess the potential impact of RoB in individual studies on the results of the meta-analysis or other evidence synthesis? 13. Did the review authors account for RoB in individual studies when interpreting/discussing the results of the review? 14. Did the review authors provide a satisfactory explanation for, and discussion of, any heterogeneity observed in the results of the review? 15. If they performed quantitative synthesis, did the review authors carry out an adequate investigation of publication bias (small study bias) and discuss its likely impact on the results of the review? 16. Did the review authors report any potential sources of conflict of interest, including any funding they received for conducting the review? Each question was answered with “Yes,” “Partial Yes” or “No”. When no meta-analysis was done, question 11, 12 and 15 were answered with “No” meta-analysis conducted.

**TABLE 3:** Summary of findings and quality of evidence assessment using the GRADE approach.

Outcome measure	Summary of findings		Quality of evidence assessment (GRADE)					
	No of patients (meta-analyses)	Effect size (95% CI)	Risk of bias^a^	Inconsistency^b^	Indirectness^c^	Imprecision^d^	Publication bias^e^	Quality of evidence^f^
**Anthropometric measures**								
BMI (kg/m^2^)	7,507 (7)	−0.11 (−0.18, −0.05)	Not serious	Not serious	Serious	Not serious	Not serious	Moderate
Body weight (kg)	15,256 (14)	−0.16 (−0.36, 0.04)	Not serious	Not serious	Serious	Not serious	Not serious	Moderate
WC (cm)	2,161 (5)	−0.79 (−1.20, −0.37)	Not serious	Not serious	Serious	Not serious	Not serious	Moderate
Fat mass	3,185 (4)	0.02 (−0.20, 0.24)	Not serious	Not serious	Serious	Not serious	Not serious	Moderate

BMI, body mass index; WC, waist circumference. ^a^Risk of bias based on the AMSTAR results.

^b^Downgraded if there was a substantial unexplained heterogeneity (I^2^ > 50%, P < 0.10) that was unexplained by meta-regression or subgroup analyses.

^c^Downgraded if there were factors present relating to the participants, interventions, or outcomes that limited the generalizability of the results.

^d^Downgraded if the 95% confidence interval (95% CI) crossed the minimally important difference (MID) for benefit or harm. MIDs used for each outcome were: 0.2 kg/m^2^ for BMI, and 2 cm for WC, 5–10% for body weight (38).

^e^Downgraded if there was an evidence of publication bias using funnel plot.

^f^Since all included studies were meta-analyses of randomized clinical trials, the certainty of the evidence was graded as high for all outcomes by default and then downgraded based on prespecified criteria. Quality was graded as high, moderate, low, very low.

### Impact of vitamin D on body weight

**FIGURE 2:**
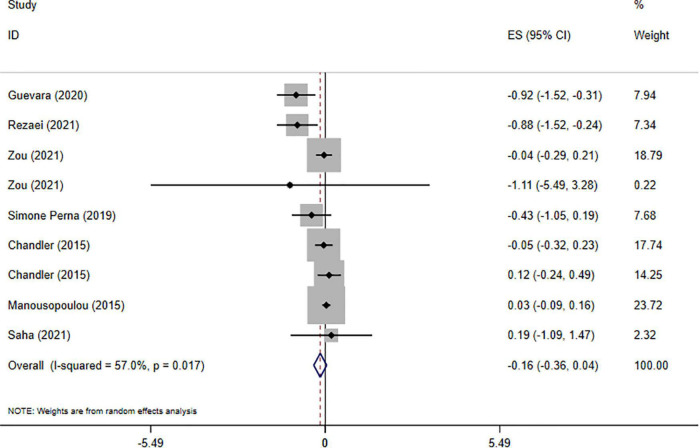
The effects of vitamin D supplementation on body weight are depicted in a forest plot with mean differences and 95 percent confidence intervals (CIs).

Overall, seven meta-analyses with nine effect sizes comprising a total of 7,507 participants have investigated the impact of vitamin D supplementation on BW levels (Figure 2). Combining their findings using random-effects model, it was found that BW was not significantly affected after vitamin D supplementation (ES = −0.16 kg, 95% CI: −0.36, 0.04; *p* = 0.125, *I*^2^ = 57.0%, *p* = 0.017). Also, the findings of subgroup analysis implied that mean age of participants, dosage, sample size, study population, and duration of intervention were the sources of heterogeneity (Table 4). No significant difference was observed performing sensitivity analysis. No significant publication bias was observed performing Begg’s test (*P* = 0.251).

**TABLE 4:** Subgroup analyses for the effects of vitamin D supplementation on obesity indices.

	Effect size, *n*	ES (95% CI)^a^	*p*-within^b^	*I*^2^ (%)^c^	P-heterogeneity^d^
**Vitamin D on BMI**					
Overall	16	−0.11 (−0.18, −0.05)	<0.001	61.0	<0.001
**Age (years)**					
≤40	5	−0.07 (−0.23, 0.08)	0.361	56.3	0.057
40–50	5	−0.15 (−0.30, 0.0)	0.050	79.3	<0.001
>50	4	−0.07 (−0.14, −0.01)	0.034	24.9	0.262
NR	2	−0.29 (−0.46, −0.12)	0.001	0.0	0.521
**Gender**					
women	4	0.0 (−0.17, 0.16)	0.965	13.0	0.327
Both	12	−0.13 (−0.20, −0.06)	<0.001	67.3	<0.001
**Intervention duration (week)**					
≤16 >16	9 7	−0.19 (−0.33, −0.05) −0.05 (−0.10, −0.01)	0.006 0.015	66.7 0.0	0.002 0.736
**Dosage (IU)**					
≤5,000	8	−0.05 (−0.09, −0.0)	0.047	0.0	0.607
>5,000	7	−0.14 (−0.23, −0.05)	<0.001	46.1	0.084
NR	1	−0.52 (−0.73, −0.31)	0.001	−	−
**Study population**					
Overweight and obesity	7	−0.12 (−0.22, −0.02)	0.019	78.0	<0.001
PCOS	2	0.03 (−0.14, 0.21)	0.699	0.0	0.480
NAFLD	2	−0.32 (−0.51, −0.12)	<0.001	0.0	0.778
Diabetes	5	−0.12 (−0.18, −0.05)	0.001	0.0	0.645
**Sample size**					
≤1,000 >1,000	9 6	−0.15 (−0.25, −0.05) −0.05 (−0.09, −0.01)	0.004 0.021	38.8 0.0	0.109 0.676
NR	1	−0.52 (−0.73, −0.31)	0.001	−	−
**Type of effect size**					
WMD	9	−0.16 (−0.26, −0.06)	0.001	73.9	<0.001
SMD	7	−0.05 (−0.11, 0.01)	0.117	0.0	0.631
**Vitamin D on body weight**					
Overall	9	−0.16 (−0.36, 0.04)	0.125	57.0	0.017
**Age (years)**					
≤50	4	−0.31 (−0.83, 0.22)	0.250	72.6	0.012
>50	4	−0.01 (−0.18, 0.15)	0.882	0.0	0.834
NR	1	−0.88 (−1.52, −0.24)	0.007	−	−
**Intervention duration (week)**					
≤16	4	−0.46 (−1.04, 0.12)	0.123	74.1	0.009
>16	5	0.01 (−0.10, 0.12)	0.816	0.0	0.585
**Dosage (IU)**					
≤5,000	3	0.02 (−0.20, 0.23)	0.880	0.0	0.739
5,000–10,000	4	−0.36 (−0.91, 0.18)	0.191	68.5	0.023
>10,000	1	−0.04 (−0.29, 0.21)	0.754	−	−
NR	1	−0.92 (−1.53, −0.32)	0.003	−	−
**Study population**					
Obesity	5	−0.13 (−0.38, 0.12)	0.303	65.1	0.022
NAFLD	1	−0.88 (−1.52, −0.24)	0.007	−	−
Diabetes	3	−0.03 (−0.28, 0.21)	0.780	0.0	0.839
**Sample size**					
≤1,000	5	−0.31 (−0.72, 0.09)	0.130	42.2	0.140
>1,000	3	0.03 (−0.08, 0.14)	0.624	0.0	0.756
NR	1	−0.92 (−1.53, −0.32)	0.003	−	−

ES, Effect size; CI, confidence interval.

^a^Obtained from the Random-effects model.

^b^Refers to the mean (95% CI).

^c^Inconsistency, percentage of variation across studies due to heterogeneity.

^d^Obtained from the Q-test. NR, Not reported; NAFLD, Non-alcoholic fatty liver disease.

### Impact of vitamin D on body mass index

**FIGURE 3:**
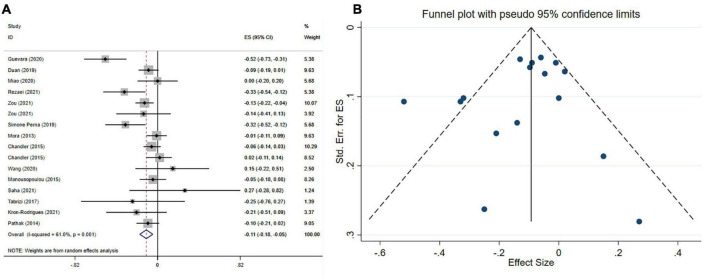
Forest plot **(A)** funnel plot with a mean difference and 95% confidence intervals (CIs) **(B)** publication bias in the studies reporting, the effects of vitamin D supplementation on BMI levels.

The effect of vitamin D supplementation on BMI was assessed in 14 meta-analyses with 15 effect sizes, including 15,256 participants. The pooled estimate indicated that in subjects who consumed vitamin D supplements, BMI significantly was decreased (ES = −0.11 kg/m^2^; 95% CI: −0.18, −0.05 *p* < 0.001) (Figure 3A). Furthermore, a high between-study heterogeneity was observed (*I*^2^ = 61.0%, *p* < 0.001), which was decreased with subgrouping according to the study population, mean age, type of effect size, sample size, treatment dosage, and duration (Table 4). Vitamin D supplement > 5,000 IU/day, intervention duration of ≤ 16 weeks and supplementation on subjects with NAFLD contributed to a greater reduction in BMI (Table 4). There was no significant difference with removing one single study using sensitivity analysis. Based on Begg’s, and Egger’s tests, no significant small-study effect has been detected (*p* = 0.718, and *p* = 384, respectively). Moreover, an uneven distribution of studies was observed after visual inspection of the funnel plot (Figure 3B). Therefore, trim and fill analysis was performed (no imputed studies) and the findings were still significant (ES = −0.11 kg/m^2^; 95% CI: −0.18, −0.05, *p*? 0.05).

### Impact of vitamin D on waist circumference

**FIGURE 4:**
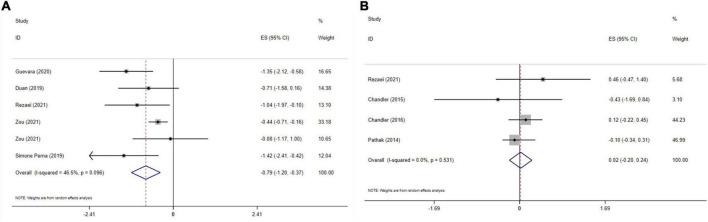
The effects of vitamin D supplementation on WC **(A)**, and fat mass **(B)** are depicted in a forest plot with mean differences and 95 percent confidence intervals (CIs).

Five meta-analyses with six effect sizes, including 2,161 participants have evaluated the effect of vitamin D administration on WC levels. The obtained pooled effect size revealed that vitamin D meaningfully reduced WC (ES = −0.79 cm; 95% CI: −1.20, −0.37; *p* < 0.001) (Figure 4A). No significant heterogeneity was detected among studies (*I*^2^ = 46.5%, *p* = 0.096). The effect size was not affected by sensitivity analysis. Begg’s test has indicated no significant publication bias (*p* = 0.999).

### Impact of vitamin D on fat mass

The effect of vitamin D on fat mass is presented in Figure 4B. Combining four effect sizes and total of 3,185 participants demonstrated that vitamin D had no significant impact on fat mass (ES: 0.02, 95% CI: −0.20, 0.24, *p* = 0.868). There was no significant heterogeneity among studies (*I*^2^ = 0.0%, *p* = 0.531). Sensitivity analysis showed that no study affected the overall effect size. Based on Begg’s test, no significant publication bias was detected (*p* = 0.734).

## Discussion

The present umbrella meta-analysis on the effect of vitamin D treatment on BW, BMI, fat mass and WC summarized the results of 14 meta-analyses including 116 trials. The findings of this study lend support to the theory that vitamin D supplementation was efficient in declining BW and BMI, whilst no significant associations were observed regarding FM and WC. Intervention duration of ≤ 16 weeks, administered dosage of > 5,000 IU, mean age of > 50 years and study population (overweight, obesity, NAFLD, diabetes) for both genders led to more improvements in BMI.

There is a bidirectional relationship between obesity and the metabolism and storage of vitamin D (29). According to observational studies, the risk of vitamin D deficiency (VDD) among obese individuals is high; however, the causality direction of this relationship is indistinct. Hence, it is not clear whether VDD is the cause or result of obesity (34). Four main mechanisms have been mentioned for the low vitamin D level in obese subjects: First, the less sun exposure compared to healthy people, since obese individuals usually avoid outdoor activity (35). Vitamin D ingested by food or synthesized in the skin undergoes two important hydroxylation stages. In the liver, vitamin D is transformed to 25(OH)D3, and in the liver, 25(OH)D3 is synthesized into 1,25 (OH) 2D. The active form of vitamin D after binding to its receptor forms a heterodimer with retinoid X receptor (RXR) and translocate into the nucleus. The so called VDR-RXR complex interacts with specific DNA regions and regulates multiple genes such as CYP27B1 expression in pancreases beta-cells (36). VDR polymorphisms display a negative role in obesity (35). Second, the high concentration of 1, 25 (OH)D in obese individuals declines the concentration of 25 (OH)D. Third, vitamin D is dispossessed inside the adipose tissue. Fourth, the volumetric dilution of fat mass, declines 25 (OH)D concentration (35). VDD and abundance of fat accumulation, together decrease the activity of alpha-hydroxylase, the main enzyme responsible for the biotransformation of calciferol in the liver. This phenomenon results in the accumulation of inactive forms of vitamin D and reduction of its bioavailability, since abdominal fat is a good storage site for vitamin D (24, 36).

VDD influences the risk of obesity by directly elevating adipogenesis or indirectly modulating inflammation, oxidative stress, metabolism, differentiation of preadipocytes into adipocytes, and gene regulation (24). VDD enhances parathyroid hormone (PTH) level and increases the invasion of calcium into adipocyte tissue and furthermore enhances lipogenesis, stimulating catecholamine to induce lipolysis and accumulate fat in adipocytes (17, 24). PTH mediates this action *via* a sympathetic nervous system thermogenesis and lipolysis mechanism (37). Evidence suggests that supplementing the active form of vitamin D, may alleviate substrate oxidation, improve insulin sensitivity, suppress PTH, promote adiponectin secretion and eventually stimulate weight loss (28). Vitamin D effects insulin sensitivity by enhancing the expression of insulin receptor in peripheral cells *via* a Ca^2+^-dependent mechanism, since vitamin D is responsible for the regulation and passage of intracellular Ca^2+^ (36). Vitamin D also influences adipocyte apoptosis, adipogenesis regulation, lipid metabolism, calcium absorption, and is associated with β cell function and/or insulin resistance (27, 29, 33).

**FIGURE 5:**
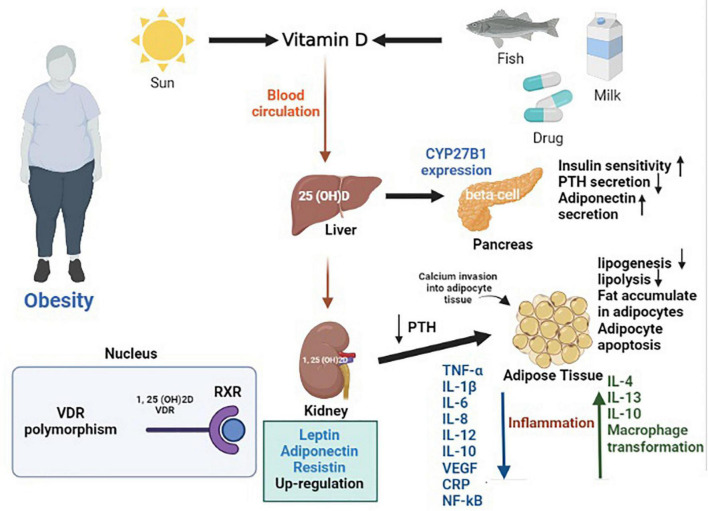
The mechanism of action of vitamin D in obesity.

Today obesity has been recognized a state of chronic, low-grade systemic inflammation. Within this situation, adipocytes secrete pro and anti-inflammatory cytokines, hormones, and acute phase reactants. Other inflammatory molecules such as preadipocytes, mast cells, and macrophages are also responsible for enhancing inflammation in obese subjects. Vitamin D acts as an acute phase reactant in the inflammatory situation caused by obesity and suppress 25(OH)D concentration. Vitamin D has a positive correlation with the adiponectin hormone in obese subjects. Thus, enhancing 25(OH)D concentration, helps increase the process of weight loss by declining inflammation (35). Additionally, 1,25(OH)2D regulates the expression of adipokines in visceral adipose tissue by up-regulating the expression of genes responsible in the secretion of leptin, adiponectin, tumor necrosis factor (TNF-alpha), plasminogen activator inhibitor type I, transforming growth factor (TGF) type I, and resistin (36). Vitamin D 1,25 (OH) 2D inhibits proinflammatory cytokines such as IL-1b, IL-6, IL-8, IL-12, Vascular endothelial growth factor (VEGF), C-reactive protein (CRP), and Nuclear factor kappa B (NF-kB) and mitogen activated protein kinase signaling pathways and reduce the expression of toll-like receptors. Also, the active form of vitamin D increases IL-4, IL-13, IL-10, and macrophage transformation (36). Hence, vitamin D is efficient in declining weight *via* controlling inflammation caused obesity. Figure 5 displays the mechanism of action of vitamin D in obesity.

Although the effect of vitamin D on WC and FM were assessed in few studies, most of the studies did not observe beneficial results. The diverse results claimed for body fat may be due to the various fat mass measures used (truncal fat, whole-body fat, and body fat percentage) instead of standard methods such as dual-energy X-ray absorptiometry (DXA) or the age of study participants since fat mass alleviates as individuals become older (29, 31). Moreover, the differences in doses of vitamin D administered and differences in serum level of vitamin D are mentioned as reason for these findings (27). However, the limited number of studies assessing the effect of vitamin D on WC and FM could be mentioned as an important reason for the insignificant results observed.

Based on subgroup analyses, vitamin D supplementation was more efficient in declining BMI when administered for individuals older than 50 years. However, since BMI is influenced by age, BMI decline may be due to other reasons rather than vitamin D supplementation. In older individuals, fat mass is increased and lean body mass and skeletal body mass are declined. Thus, these factors are also explanations for the reduction of BMI in older subjects (29). In regards to gender differences, according to Table 4, the number of studies assessing the effect of vitamin D supplementation on BMI in females were low and the majority of studies had matched basic characteristics. Thus, although gender does impact one’s ability to lose weight (28) along with hormonal changes in the body such as menopause (32), subgroup analyses indicated beneficial effects for vitamin D supplementation in both genders. This could be due to the high prevalence of vitamin D deficiency in both genders and the effectiveness of vitamin D in compensating deficiencies in VDD subjects. Subgroup analyses for duration of intervention indicated significant results for both cut-offs (Table 4). This may be because the majority of studies had not claimed how long each study had maintained their achieved level of 25(OH)D. Also, while vitamin D administration for short term can normalize serum levels, cessation of supplementation may affect the retention of beneficial effects (28). Additionally, vitamin D supplementation should be applied for a certain timing in order to observe significant changes in BMI (24). Similar findings were also observed regarding vitamin D dosage. The differences in serum levels of vitamin D is an important factor affecting the appropriate dose of vitamin D. Moreover, adequate response to vitamin D is inversely associated with baseline BMI, thus body size should be deliberated when determining suitable dose of supplementation (27). In overweight and obese subjects, as well as patients diagnosed with NAFLD and T2DM, the percentage of central fat is high, subjects have a sensitive response to supplementation and easily display the beneficial effects of vitamin D. Hence, vitamin D administration is efficient in declining BMI in this group of patients (24). However, the insignificant results for PCOS patients may be due to the limited number of studies, poor methodological biases reported, small sample size, short duration of intervention, and variations in doses and units of vitamin D (20, 25).

Subgroup analyses based on the effect of vitamin D supplementation on body weight indicated significant findings regarding NAFLD patients; however, it was only applied in one study which makes judgment partially difficult. In Rezaei et al.’s study, the majority of studies were accomplished in Iran with similar basic characteristics; therefore, the results of this meta-analysis could not be generalized to the whole population (27).

The most notable strength of this umbrella meta-analysis was performing sub-group analyses, controlling publication bias and conducting comprehensive search of the literature. The majority of the included studies reported that more than half of the performed RCTs were methodologically qualified according to Cochrane, Jadad, and SIGN tools. Also, the current review was registered in PROSPERO or Cochrane library. There were few limitations that must be noted as well. First, the significant between-study heterogeneity reported among studies which was reduced in certain subgroups. Second, the various range of study populations with different characteristics. Third, the included studies were accomplished in certain geographic regions (mostly Asian regions) which may have enhanced the possibility of selection bias. Forth, omitting the effect of environmental factors such as sunlight or diet on serum 25(OH)D status.

## Conclusion

The present umbrella meta-analysis confirms the potential benefits of vitamin D supplementation in reducing anthropometric indices such as BMI and BW, but not WC and fat mass. Moreover, vitamin D supplementation with a dosage of > 5,000 on overweight and obese subjects, NAFLD and diabetic patients, subjects older than 50 years and with intervention duration ≤ 16 weeks contribute to a more pronounced influence in lowering BMI. In this regards, vitamin D could be administered as a complementary treatment in the management of overweight and/or obesity.

## Data availability statement

The original contributions presented in this study are included in the article/Supplementary material, further inquiries can be directed to the corresponding author/s.

## Author contributions

VM, FG, and MZ designed the research and wrote the manuscript. VM and FG conducted the systematic search. VM and FK screened the articles and extracted the data. VM and MZ analyzed and interpreted the data. FG and FK drew the tables. ZG and MZ had primary responsibility for the final content. All authors read and approved the final manuscript.
