# Supplementary material for: Vitamin D supplementation positively affects anthropometric indices: Evidence obtained from an umbrella meta-analysis
Source: Front Nutr. 2022 Sep 7;9:980749. doi: 10.3389/fnut.2022.980749 (PMC9490226; doi:10.3389/fnut.2022.980749)
Supplement: Supplementary file 1 [file Table_1.DOCX]

**Based on MeSH and text keywords, the following pattern of search was applied:**

| "vitamin d"[MeSH Terms] OR "ergocalciferols"[MeSH Terms] OR "vitamin d"[MeSH Terms] OR "vitamin d"[All Fields] OR "ergocalciferols"[MeSH Terms] OR "ergocalciferols"[All Fields]OR"treatmen"[Title/Abstract] OR "supplementation [Title/Abstract] OR "vitamin d3"[Title/Abstract] OR "vitamin d2 [Title/Abstract] OR "intake"[Title/Abstract]) **AND** "body weight" [Title/Abstract] OR "body weight changes" [Title/Abstract] OR "body mass index" [Title/Abstract] OR "weight loss" [Title/Abstract] OR "obesity" [Title/Abstract] OR "body weight" [Title/Abstract] OR "body mass index" [Title/Abstract] OR "BMI" [Title/Abstract] OR "waist circumference" [Title/Abstract] OR "WC" [Title/Abstract] OR fat mass [Title/Abstract] **AND** "systematic review" [Publication Type] OR "meta-analysis" [Title/Abstract]. |
| --- |
